# Exploring the Role of Nurses in Advance Care Planning Within Long-Term Care Homes: A Qualitative Study

**DOI:** 10.1177/23779608241249335

**Published:** 2024-04-29

**Authors:** Harveer Punia, Sharon Kaasalainen, Jenny Ploeg, Patricia Strachan, Tamara Sussman

**Affiliations:** 1 School of Nursing, 3710 McMaster University, Hamilton, Canada; 2 School of Social Work, 5620 McGill University, Montreal, Canada

**Keywords:** advance care planning, long-Term care homes, nursing homes, nursing

## Abstract

**Background:**

Residents in long-term care homes (LTCHs) are often diagnosed with chronic, life-limiting illnesses, and it is now a common site to provide high levels of care and eventual death. There is an urgent need to address communication gaps and uncertainties surrounding resident's end of life preferences. Nurses are well situated to be key facilitators of necessary advance care planning (ACP), ensuring residents have discussions with family, substitute decision-makers and healthcare providers regarding future health and personal care preferences. However, LTCHs present unique challenges for nurses due to not only complex comorbidities but also staffing dynamics.

**Purpose:**

This study explored the experiences and perceptions of Registered Nurses (RNs) and Registered Practical Nurse (RPNs) in LTCHs regarding their role in engaging residents and families in ACP discussions.

**Methods:**

Qualitative interpretive descriptive methodology was used. Data were collected from two LTCHs in Southern Ontario with a sample of 15 nurses (7 RNs and 8 RPNs). Analysis involved review of semistructured interviews, field notes, and utilizing constant comparison within an inductive approach.

**Results:**

Power and authority dynamics in LTCH's was an overarching theme in the data, with four subthemes: (1) Nurses lacking clarity about ACP, (2) nurses’ uncertainty regarding their role in ACP, (3) nurses feeling uncomfortable engaging in ACP discussions, and (4) nurses struggling to support families in ACP discussions.

**Conclusion:**

Recommendations for nurses, healthcare providers, LTCH administrators, and policy makers include: (1) development of policies which support, from a systemic level, nurses to feel safe while engaging in ACP; (2) reassessing LTCH's hierarchical structure, and clarifying RN, RPN, and interdisciplinary team members roles in ACP; (3) developing culture change that allows a team and person-centered approach to ACP; and (4) providing ongoing education and mentorship for nurses to manage family dynamics and expand their understanding of ACP beyond a biomedical lens.

## Introduction

The aging population has driven the demand for long-term care homes (LTCH) in Ontario, with a projected 10-fold increase by 2038 (Canadian Institute for Health Information [CIHI], 2017; Canadian Nurses Association [CNA], 2016a). A report from 2021 to 2022 identifies that over 6,000 residents of Canadians LTCHs were transferred to and subsequently died in hospital (CIHI, 2023). As more residents live in LTCHs and on-site deaths rise (Hirdes et al., 2011; Marcella & Kelley, 2015), there is a pressing need to address communication gaps and uncertainties surrounding resident's end of life (EOL) care preferences.

A palliative approach to care in LTCHs aims to enhance quality of life (QOL) for individuals diagnosed with life-limiting chronic illnesses. It encompasses advance care planning (ACP), symptom management, supportive care, EOL care, and bereavement (Sawatzky et al., 2016). ACP encourages individuals to express care preferences, ensuring healthcare providers, substitute decision-makers, and family members, understand their values should they lose decision-making capacity later in their illness trajectory (Beck et al., 2017; Canadian Hospice Palliative Care Association [CHPCA], 2020). Given that LTCH residents’ preferences for EOL care are often unknown, ACP becomes especially beneficial in facilitating and influencing care decisions in this setting (Ransbottom & Kelley, 2014). The consequence of such oversight can lead to providing care that does not align with residents’ wishes, undesirable hospital admissions and invasive medical interventions, negatively impacting both the residents’ QOL and the healthcare system (Ransbottom & Kelley, 2014).

### Staffing Structure in LTCHs

Nurses alongside unregulated workers (e.g., personal support workers, and healthcare aides) comprise the largest occupational groups providing care to LTCH residents. The staffing mix in LTCHs, where on-site physicians and Advanced Practice Nurses (Nurse Practitioners and Clinical Nurse Specialists) are rarely on site, places a heavy reliance on Registered Nurses (RNs) and Registered Practical Nurses (RPNs) to coordinate safe and competent care for residents (OLTCA, 2018). Despite RNs having a broader scope of practice, in reality they account for only 9% of all healthcare providers in LTCH settings, while RPNs account for 17% (Ontario Association of Non-Profit Homes and Services for Seniors [OANHSS], 2015). Given the limited availability of RNs, a preferred model of care delivery would reflect one where both RNs and RPNs work collaboratively with other healthcare professionals, and to their full scope of practice to optimize the care provided to residents (Canadian Nurses Association [CNA], 2013). The professional responsibility of nurses, as outlined by the Canadian Nurses Association (2013), is to encourage individuals to engage in ACP. While both subgroups of nurses appear well situated to play a significant role in engaging residents and their families in ACP, there is a need to explore how they perceive their role in this process.

### Review of Literature

#### ACP in LTCH

A growing body of research suggests ACP has positive benefits for residents of LTCHs. It enhances communication and increases familiarity between residents, their healthcare team, and families (Cornally et al., 2015; Robinson et al., 2011; Shanley et al., 2011; Stewart et al., 2011). It also has been well known that residents prefer to stay in LTCHs during EOL care and through engaging in ACP, a reduction in unwanted hospitalization and avoidance of crisis decision making can be achieved (Cornally et al., 2015; Fernandes, 2008; Robinson et al., 2011; Shanley et al., 2011).

Despite the known benefits of ACP, it is not a common practice in LTCHs and has been found to be rarely implemented or observed (Butler et al., 2014; Ong et al., 2011). Literature indicates that engaging in ACP is a challenge for staff and families of residents especially when the resident has moderate to severe cognitive impairments. LTCH staff including nurses and managers face barriers and uncertainty about how to engage in ACP with residents with dementia and variable levels of cognitive impairments (Ampe et al., 2015, 2017; Cornally et al., 2015; McGlade et al., 2017; Stewart et al., 2011; Thoresen et al., 2019).

#### Nurses’ Role in ACP in LTC

A body of research is emerging suggesting that more targeted education is required for nurses to better understand the process of ACP (Beck et al., 2017; Gilissen et al., 2017; Hickman et al., 2016). A systematic review conducted by Gilissen et al. (2017) identified numerous barriers hindering the successful implementation of ACP in LTCHs. One key barrier was the need for LTCH staff, including nurses, to better understand attitudes, roles, and skills related to ACP to successfully incorporate it into the care process (Gilissen et al., 2017). Similarly, it has been highlighted that deficits related to knowledge and education result in low compliance and uptake of ACP by staff (Beck et al., 2017; Fernandes, 2008; Flo et al., 2016; Thoresen et al., 2016).

Although nurses have a crucial role in care provided in LTCHs, there is a paucity of literature exploring their perceptions and experience in respect to their role in engaging in ACP (Gilissen et al., 2017; Li-Shan et al., 2015; Ng & Wong, 2021). It was generally found that LTCH nurses were unclear about their role in ACP and also felt families did not completely understand the nurses’ role (Beck et al., 2017; Handley et al., 2014; van Soest-Poortvliet et al., 2015). Previous research has not addressed this topic in-depth, and although some findings may be transferable to the nurses’ role in ACP in Canadian LTCHs (Beck et al., 2017; Fernandes, 2008; Flo et al., 2016; Gilissen et al., 2017; Thoresen et al., 2016), studies conducted in the Canadian context are scant. The legality and scope of practice of nurses may significantly vary from other settings (CHPCA, 2020).

Furthermore, there is a lack of research that explores the experiences and perceptions of both RN and RPNs. There is a critical and urgent need to describe and interpret the experiences of both subgroups of nurses and clarify their role in implementing ACP. Therefore, this study addressed the following research question: What are the experiences (e.g., observed and lived in their practice) and perceptions (e.g., awareness and beliefs) of both RNs and RPNs in LTCHs with respect to their role in engaging residents and families in ACP discussions.

## Methods

### Design

The experiences and perceptions of nurses working in LTCHs with respect to their role in ACP was explored through Interpretive Description (ID) methodology. ID was appropriate to use for the research study because through both description and rich interpretation of experiences, the uniqueness of each individual experience is acknowledged and can be applied back to the LTCH context (Thorne, 2016).

### Setting and Sample

Data was collected from nurses at two LTCHs in a city in Southwestern Ontario, Canada. The first LTCH is a 127 bed private owned facility while the second is a 160 bed, not-for-profit, government owned LTCH. Recruitment took place between May 2019 and September 2019. Inclusion criteria ensured participants were English-speaking LTCH nurses who provided or oversaw care for residents with chronic illnesses in the past six months. Two types of nurses were intentionally recruited; RNs and RPNs. The College of Nurses [CNO] (2018) emphasizes that RPNs’ education focuses on patients with stable, and less complex needs, while RNs obtain greater foundational knowledge in practice, critical thinking, leadership due to their higher level of education. Factors of complexity, predictability, and risk of negative resident outcomes are used to determine how an RN or RPN is utilized within LTCHs. Thus, because of the differences in their scope of practice the specific roles nurses take differs across LTCHs.

The recruitment strategy involved a combination of purposive sampling techniques and snowball sampling. Contacting management at both LTCH sites via email or telephone was the first step, followed by meetings to confirm their interest and seek permission to display recruitment posters. Management recommended potential participants who met inclusion criteria, and the researcher shared study information with them and answered any questions. Written consent was obtained and a total of 15 participants (7 RNs and 8 RPNs) were included. The researcher ensured there was a balanced representation of demographic factors such as age, years of experience, and employment type. The primary researcher identifies as a female, bachelor's prepared RN with acute care experience. The primary researcher had no previous relationships with the study participants.

### Data Collection Procedures

Semistructured interviews were conducted in person or via telephone/web-based platforms, lasting 30–60 min with the primary researcher. Pilot interviews with two nursing colleagues were also conducted. Participants were able to choose the time and location of interviews to ensure privacy and confidentiality. Most interviews were conducted at participants’ place of work, except two participants who chose to be interviewed over telephone. Demographic information was collected and interviews were audio recorded, transcribed, and supplemented with field notes. When the interview was concluding, the researcher ensured a summary of key points was shared, and allowed participants the opportunity for clarification. None of the participants dropped out. Gift cards were offered to participants as incentives ($25.00) and for participation in research.

Thorne (2016) argues that relying solely on the concept of data saturation is inadequate for determining sample size and concluding analysis, especially in the health sciences where participants often exhibit infinite variations of experiences. Data collection concluded when the research team agreed that the clinical phenomenon had been sufficiently explored, as assessed through the quality of collected data (Guetterman, 2015; Thorne, 2016).

### Data Analysis

Data collection and analysis in this qualitative study was an iterative process. All semistructured interviews were transcribed and re-checked the transcripts against the audio recording to ensure accuracy. An inductive data analysis approach was used and initiated by the primary researcher by reading and re-reading field notes and transcripts to familiarize with the data (prior to commencing the coding process) (Thorne, 2016).

A line-by-line hand coding process was used for all transcripts. The strategy of constant comparison was used where the primary researcher was consistently comparing results across participants, exploring emerging patterns and adjusting the interview guide accordingly to deeper explore patterns or themes if necessary. In order to develop the preliminary coding scheme, the primary researcher and an additional researcher coded the first three transcripts. Those remaining were coded solely by the primary researcher.

With 15 interviews, recurring patterns were identified; preliminary findings were discussed with all authors, recruitment was stopped, and the findings were synthesized and applied to the practice of LTCH nurses. To enhance credibility, rigor, and trustworthiness of findings, several strategies were implemented. The primary researcher maintained a reflexive and analytical journal to acknowledge and document intrinsic biases and preconceptions regarding the phenomenon of interest (Creswell & Poth, 2018; Lincoln and Guba, 1985; Thorne, 2016). Investigator and data source triangulation was also established by recruiting both RNs and RPNs and employing various data collection methods (i.e., semistructured interviews, field notes, and reflexive journaling) (Sandelowski, 1995; Thorne, 2016).

## Findings

### Sample Characteristics

The study sample consisted of 15 nurses working in two different LTCH settings (see Appendix Table 1) for participant demographics). Of the 15 nurse participants, seven were RNs (47.0%) and eight were RPNs (53.0%); with 93.3% of the total sample being female. The average age of nurses was 47.8 years (SD 12.1), with ages ranging from 25 to 60 years of age. The mean number of years worked in the LTCH setting was 15.0 (SD 9.2) and ranged from 2 to 25 years. Most nurses had not received any training regarding ACP (60.0%, n  =  9). Of the nurses that had been trained in ACP, one participant had received it within the past 6 months and 5 had received it over a year ago. The format of training varied from a previous course (n  =  3), informational brochure (n  =  1), presentation (n  =  1) or general content obtained in undergraduate education (n  =  1).

**Table 1.** Characteristics of Study Participants.

Variable	n = 15 (%)
Sex	
Male	1 (6.7)
Female	14 (93.3)
Profession	
Registered nurse	7 (47.0)
Registered practical nurse	8 (53.0)
Employment status	
Part-time	1 (6.7)
Full-time	13 (86.7)
Casual	1 (6.7)
Training in ACP received?	
Yes	6 (40.0)
No	9 (60.0)
How recently was ACP training received?	
Within a month	0 (0)
Within the past 6 months	1 (6.7)
Within a year	0 (0)
Over a year ago	4 (26.7)
Not applicable	10 (66.7)
Format of ACP training	
Brochure	1 (6.7)
Course	3 (20.0)
Other	2 (13.3)
Not applicable	9 (60.0)
	Mean (SD)
Age	47.8 (12.1)
Years worked in LTCH	19 (9.2)
Years worked in current LTCH facility	15.7 (9.1)

*Note. N* = 15. Data collected by author from June–September 2019. ACP=advance care planning; LTCH=long-term care home.

### Overarching Theme: Power and Authority Dynamics in LTCHs

The analysis revealed that the organizational structure and context of LTCHs influenced the perceptions and experiences of both RNs and RPNs regarding their role in facilitating and engaging in ACP. The perception of hierarchical staffing, where power was concentrated at top leadership levels, hindered their ability to initiate and engage in holistic ACP with families and residents. An RN shares,

“According to our facility and policies we need to have a DNR (do not resuscitate) or CPR (cardiopulmonary resuscitation) status in 24 hours. I try to explain [ACP] to the best of my understanding, but I really do not have any option to explain more.” (Participant 7, RN)

One RPN acknowledged that although nurses may appear to be in an ideal position for ACP with residents, the structure of LTCHs does not currently optimize their role or ability to participate in ACP conversations.

“There is [a role for RPN's in ACP] but it's just the hierarchy here…as RPNs it's not really expected of us. More, when someone starts to go we usually call the RN and say the family is here we need to call the doctor.”(Participant 12, RPN)

Another RPN further emphasizes the lack of a team approach to engage in ACP due to power dynamics. Specifically, the deference to physician authority, and feeling constrained is highlighted:

“We have closer contact with the resident on a daily basis, versus the doctor, but I think the doctor has more authority”… for example “Voicing I can’t do anymore treatment…” as a nurse, I don’t think we could say that [to the family].” (Participant 14, RPN)

It appears that both subgroups of nurses identify ACP as equivalent to discussing prognosis or medical decision-making conversations that they do not feel authorized or encouraged to engage in. However, RPNs highlighted the absence of a team-based approach where both subgroups of nurses and physicians collaborate. This may have contributed to RPNs particularly feeling disempowered to be part of ACP.

### Theme 1: Lacking Clarity About ACP

This theme highlights the uncertainty both subgroups of nurses felt regarding what the content and process of ACP is. Most participants’ views regarding ACP were restricted to the decision-making process of a resident's code status. Participant 4 (RN) stated, “*The advanced care plan we have here is the DNR or the full code—is that what you're looking at?”* Frequently nurses also interpreted ACP as an isolated event, to complete medical decision making and a legal advance care directive.

“…On the first day we discuss palliative care…they have to sign a paper which is the advanced care directive and that will be placed on the resident's chart for our facility…ACP is like introducing initiatives, do not resuscitate DNR or CPR…” (Participant 9, RPN)

Another RN shared their understanding of ACP:

“So, um, advanced care planning is specifically mostly the code status. Yes or no? As far as I know… Advanced care planning is only about that…” (Participant 2, RN)

It appears both RNs and RPNs seem to have a limited understanding about ACP that does not expand a biomedical lens, and focuses on interventions near death.

### Theme 2: Uncertainty About Nursing Role in ACP

RNs and RPNs generally expressed a lack of clarity about their responsibility for ACP, and RPNs in particular exhibited a multifactorial fear of engaging in ACP discussions. Both sub groups struggled to take ownership of this role, attributing the lack of engagement to workload and perception. A participant explored why nurses may not engage in ACP discussions and rely on other professionals to take ownership. They stated, “*I think some of it is about workload and some of it is about perception as well, that that's not within my scope of practice or my realm of practice”* (Participant 1, RN).

An RPN reflected that they believed social workers were well positioned to engage residents and their families in ACP discussions:

“A social worker can [engage in ACP conversations], because that is one of their specialties. I think the social worker and the doctor have more leverage, more leeway, more authority to go in depth. We can lead the person if that person initiates conversation but at the same time you have to be careful…” (Participant 14, RPN)

One RN commented, “*The communication from the social worker is really good, and the doctors really are the ones that engage with residents. In my experience, no matter how much I explain [ACP] to the family, it is different when the doctor does it.”* It seems RNs felt physicians and social workers are perceived to have greater authority to initiate ACP. Power, authority, and opportunity all played a role in influencing both subgroups in different ways.

In contrast, the majority of RPNs believed that RNs are better suited for the role in engaging residents and families in ACP. A participant stated:

I don’t think I am [able to engage in ACP] because being a practical nurse I deal more with the resident's medications, doctors’ orders… I don’t think I would have time to just sit down and talk with the family, more RNs have that power and the director of care. (Participant 11, RPN)

Overall, both subgroups of nurses shared an uncertainty about whether engaging in ACP falls within their role and scope of practice. However, RPNs, in particular, expressed feeling concerned about overstepping boundaries. There was a prevalent perception that RNs have a broader scope of practice compared to them. As a result, it appears that ACP is not viewed as a collaborative team effort within LTCHs by both subgroups, and RPNs especially may feel that they lack the authorization to participate.

### Theme 3: Feeling Uncomfortable About Engaging in ACP Discussions

Multiple RNs explored factors that contributed to their lack of comfort with engaging residents and their families in ACP. A participant shared:

I think lacking would be our communication skills, like how would you acknowledge a family member or a resident if they said “these are the things I wanted to do, and I am passing.” I think the comfort level of nurses varies, sometimes they’re comfortable, sometimes they are not. (Participant 6, RN)

Another participant elaborated on the idea that it is their personal values which surround the topic of ACP that make it uncomfortable for nurses to engage in conversations. They state:

The big gap is the communication. And I would say the comfort as well—to be discussing it [ACP]. I think it is unavoidable on their [nurses] part for them to interject with their own perceptions, their own decision. (Participant 2, RN)

A participant provided further insight into another layer of why RNs may feel uncomfortable engaging in ACP. They stated, “*It's not like we don't know what to say. But I guess it's just being so limited to what we can say, or you know, whether we can do that”* (Participant 4, RN). While RNs may feel they have the knowledge to initiate ACP conversations on a personal level, they perceive they may not be authorized to; therefore, they struggle to begin that conversation.

Similarly, RPNs also expressed needing to feel support or protection to engage in ACP. Participant 14 (RPN) stated:

…Some clarification [scope of nurses in respect to ACP] is needed because as a nurse, you might say the wrong thing, and then the wrong thing is interpreted differently, but you have to have a protective measure.

Overall, both subgroups of nurses seemed hesitant to participate in ACP conversations. However, RNs appeared to place a greater emphasis on feeling discomfort about how to engage residents and their families in ACP conversations effectively. Majority of RPNs, on the other hand, expressed a sense of feeling unauthorized to engage in ACP. Therefore, power and authority dynamics within the LTCH context appear to influence RPNs in particular, and lead to discomfort with ACP engagement.

### Theme 4: Struggling to Support Families in ACP Discussions

Most nurses perceived that families can be challenging to engage in ACP, which may impact their ability to discuss holistic ACP and support residents. An RN discusses their experience when engaging residents and their families in ACP and expands on the potential discrepancies that can arise between them. They stated:

“The resident himself might be okay with it but then sometimes the family is not so then you have that tug of war between family and the resident that makes it difficult for you to deal with that resident. You are dealing with the resident that comes first but you also have to comfort and explain to the family what they still don’t get because they’re still in denial.” (Participant 13, RN)

Multiple nurses have the preconceived notion that families will not be receptive to ACP conversations activated by healthcare team members. A nurse reflected on the fear of upsetting families: “*Mostly it's about the family being mad. Or family—for example the substitute decision maker might have conflicting values in regard to future plan of care”* (Participant 6, RN).

Generally, it seems the fear of engaging in ACP with residents and their families appears multifactorial and present in both subgroups of nurses. An RPN expressed fear to engage resident's families in ACP discussions, due to the thought it will not be perceived as acceptable and may result in a complaint:

Because some residents will act as if they’re receptive, they like the idea, but they can still go back and complain to their family and say I’m not sure what the nurse was trying to tell me, she wants to talk to me about putting things into perspective and it's not her place. (Participant 14, RPN)

Another RN also explored the notion of feeling “safe” when engaging in ACP by stating:

“It all depends on the RN working [in management]—you can gauge who is working, who has the knowledge and communication skills and things like that [to engage in ACP]. So yes, if we are safe enough, and the family is not problematic we participate.” (Participant 2, RN)

It is possible that nurses may be inclined to avoid ACP conversations to prevent upsetting families. There is also concern about potential complaints, which can lead to tension and fear of engaging in ACP with families. Both subgroups also indicate that they require organizational support and encouragement to feel comfortable to engage in ACP conversations with residents and families.

## Discussion

### Need to Foster a Culture of Support, Collaboration and Empowerment for Nurses

One of the most significant findings of this study is that the culture of LTCHs often fails to support and empower nurses in effectively engaging residents and their families in ACP.

The hierarchical team structure directly impacted both RNs’ and RPNs’ comfort levels in activating ACP, as they both were unclear if it was their role to facilitate ACP discussions.

The current study adds to the existing body of literature by revealing the similarities and differences both subgroups of nurses experienced and perceived in respect to ACP. The perceived lack of power and authority varied for each subgroup. RNs often felt physicians and social workers had more authority to engage in ACP conversations whereas RPNs felt ACP discussions were more within the role of RNs. Notably, for RPNs, a lack of role ownership, and hesitancy to engage in holistic ACP could be associated with a perceived hierarchical structure in LTCH that does not hold healthcare providers equally accountable for ACP.

It is well known that LTCH culture and the level of support by management is a key factor that motivates staff to engage in ACP with residents (Ampe et al., 2017; Gilissen et al., 2017). A systematic review by Gilissen et al. (2017) examined the preconditions for effectively implementing ACP in LTCHs. Their systematic review found that in order for LTCH healthcare staff to engage in ACP, they need a system approach where various levels of management, stakeholders, and frontline staff are open and willing to participate in it (Gilissen et al., 2017). In order to support this approach, it is critical to foster a supportive culture in LTCHs where all levels of staff work together to put systems in place that encourage effective ACP with residents and families (Gilissen et al., 2017).

This current study highlighted the challenges faced by both RNs and RPNs due to the perceived hierarchical structure in LTCHs, and the resulting impact on their engagement in ACP. Notably, a significant finding was that nurses, particularly RPNs, exhibited reluctance to engage in ACP, primarily because they felt unauthorized to do so. They expressed that this reluctance may be partially rooted from feeling discouraged to engage in ACP conversations that they perceived could cause families and residents distress; and consequently lead to negative consequences for them from management. Overall, this study adds to the current body of literature by emphasizing that there is an urgent need to explore ways to clarify the organizational approach to ACP, and roles of both RNs and RPNs. RPNs in particular, may need support, collaboration, and leadership from RNs, in order to feel safe to activate and engage in holistic ACP with residents and families. To enable RNs to support RPNs and the team to integrate ACP into practice, both RNs and RPNs can benefit from educational and organizational support. By nurturing RNs knowledge and extending organization support, they can cultivate leadership and enhance self-efficacy essential for engaging in ACP (Gilissen et al., 2020).

### Need to Develop Nurse's Knowledge, Comfort, and Capacity to Engage in ACP

The findings of the current study are consistent with previous research that accentuates the substantial need to develop nursing knowledge, comfort and capacity to engage in holistic ACP (Ampe et al., 2015; Ong et al., 2011). Previous literature has reported a knowledge gap related to ACP in LTCH staff. A need for more education for nurses regarding the fundamental knowledge, terminology, purpose, and process of ACP was often identified (Beck et al., 2017; Fernandes, 2008; Flo et al., 2016; Gilissen et al., 2017; Hickman et al., 2016; Thoresen et al., 2016). This current study echoed similar conclusions such as nurses often used inconsistent terminology and concepts to describe ACP and lacked knowledge regarding the process. However, other new factors related to LTC nurses’ perception of ACP, added to the existing body of literature were also identified.

It is significant that both subgroups of nurses often activated and perceived ACP through a narrow and medically driven lens that was limited to code status and treatment preferences. Furthermore, on an organizational level, nurses often perceived ACP as an isolated event that took place during structured time frames (i.e., care conferences and at time of admission). Nurses were not able to identify when ACP should be initiated, which suggested that ACP is not currently supported or understood as an ongoing and holistic process in LTCHs.

This study further added that ACP from a nursing perspective may have a narrow focus on code status discussions, current care planning and medical decision making, not solely due to a knowledge gap but also due to the context of LTCHs. Nurses may perceive their role is confined to ensuring code status and medical decision making has been discussed because tasks such as ensuring code status and medical decision making is supported through current policies and documentation systems. Therefore, they may prioritize and feel responsibility to ensure those elements are discussed and documented. In the current study, RNs in particular recognized there was a need to have a more comprehensive approach to ACP, and acknowledged it is currently not part of their practice.

It is well cited that there is a lack of clarity and comfort related to how to engage residents with cognitive impairments in ACP (Beck et al., 2017; Fernandes, 2008; Flo et al., 2016; Gilissen et al., 2017). It has been reported that nurses and LTCH staff may require greater support and training regarding documentation, legality, and effectively incorporating ACP into practice (Beck et al., 2017; Gilissen et al., 2017; Fernandes, 2008; Flo et al., 2016; Thoresen et al., 2016).

This study delves deeper into the prevalent reluctance of nurses to engage in ACP. It is evident in the study fundings that this reluctance partially stems from a fear of overstepping boundaries. While both nurses acknowledged their discomfort with ACP, and how best to communicate, a majority of RPNs expressed uncertainty about whether they had the authority to broach ACP conversations with residents and their families. In order to engage residents in ACP, they often felt that they needed a protective measure. This is noteworthy, as it indicates not only the need for nurses to expand their knowledge for ACP beyond the biomedical lens, but also to the need for ACP guidelines for their role, especially with residents with cognitive impairments.

### Need for Person and Family-Centered Approach to ACP

Another significant finding from this study was that a majority of nurses were uncertain about their role in ACP. A systematic review was conducted by Li-Shan et al. (2015) to explore nurses’ views regarding implementing ACP for older adults. Their findings similarly reported nurses working in various clinical settings have conflicting views of ambiguity around whose role it is to engage residents and their families in ACP. They found some nurses believed it was within their scope of practice however did not want to be responsible, and others reported they were well positioned to engage in ACP (Li-Shan et al., 2015).

It becomes clear in the current study findings, that a lack of ownership and accountability may be the direct result of the sole medical focus given to these conversations. If nurses perceive ACP to only involve medically oriented discussions, naturally, they may overestimate the physician's role and underestimate their own. Overall, the systematic review revealed nurses perceived ACP roles to be overlapping within multidisciplinary teams adding to even more uncertainty (Li-Shan et al., 2015). However, the systematic review was not specific to nurses working in LTCH settings, therefore it is not entirely reflective of perceptions within LTCHs. The current study added to the existing body of literature since it compared the experiences and perceptions of RNs and RPNs with respect to engaging in ACP. A key finding was that RNs and RPNs perceived their roles and authority to participate in ACP to be different. Most RPNs expressed that ACP is a responsibility that should fall on RNs, as they are better prepared to engage in it. Most RPNs perceived RNs to have more authority to participate in ACP, and the RPN role was to engage in more frontline, clinically oriented tasks, and thus have less time for ACP discussions.

The current study emphasizes that LTCH organizations, and nurses in particular, require a team-based and person-centered approach to ACP to ensure residents benefit from it. Previous literature has stressed the importance of relationship-based care models in improving the quality of care for older individuals in various healthcare settings (Dewar & Nolan, 2013; McGilton et al., 2012). This approach emphasized the need to feel comfortable exploring the opinions of all parties involved, even if they may hold different beliefs. Additionally, it emphasized the importance of supporting nurses in engaging in deeper, compassion-centered conversations with patients and families (Dewar & Nolan, 2013; McGilton et al., 2012). The present study revealed that nurses may anticipate potential conflicts and differing opinions with patients and families, which may lead to hesitancy in engaging in ACP. However, in order to truly support families and residents through ACP, there needs to be an environment where open and honest discussions can thrive, which should be a shared responsibility among LTCH interdisciplinary team members, and both RNs and RPNs.

### Strengths and Limitations

To the authors’ knowledge, this is the first study to do an in-depth exploration of the experiences and perceptions of LTCH nurses with respect to their role in ACP in the Canadian setting. The inclusion of both RNs and RPNs working in LTCHs allowed for representation of the nursing role. There were also a few limitations in the study. The study was limited to two LTCHs in the geographical area of Southern Ontario. LTCH nurses working outside of this area may have different perceptions and experiences of their role in ACP, and the results may not be transferable. Lastly, in future studies, it would be useful to examine the experiences and perceptions of other healthcare providers (e.g., physicians, nurse practitioners, allied health, and PSWs) who may engage in ACP, in order to gain insight into their roles.

### Implications

The study findings have significant implications for nurses working in LTCH settings, as well as administrators and policy makers. ACP is within the scope of practice for nurses’ as outlined by the College of Nurses (CNO, 2018). However, it is clear that ACP is not being practiced holistically by nurses in LTCHs. Therefore, training and education for LTCH nurses needs to focus on expanding their understanding of ACP to go beyond the biomedical focused model of care. It is particularly important that nurses recognize the value of holistic ACP and feel confident in engaging in it. In addition to training to expand their understanding, they must also be given safe permission to move beyond discussions surrounding code status to broader values and wishes residents may have regarding personal and medical care, thereby supporting residents’ improvement in QOL at EOL.

It has previously been well supported that relationship-based approaches can improve the experience of individuals, families, and healthcare staff (Wilson, 2017). Therefore, an approach where both RNs and RPNs perceive family engagement in ACP as an important contribution is critical (Wilson, 2017). There is an urgent need for education and training for both subgroups of LTCH nurses focusing on navigating family dynamics is crucial. These offerings could be supported through the development and use of guidelines and strategies that encourage a person and family centered, team-based approach to ACP. Focused education on managing complex family dynamics can further support and encourage nurses to build comfort with ACP, and integrate engaging in holistic ACP with residents and families into their routine practice (CNO, 2018).

Lastly, it is clear that there is a need to clarify the role of various interdisciplinary team members, as well as provide strategies for collaboration in engaging residents and families in ACP. Therefore, it is recommended that LTCHs establish a comprehensive framework or guideline for ACP within their organization. Specifically, it is important to outline how RNs and RPNs can collaborate, as well as when and in what capacity can other team members support this process. This would serve to eliminate potential uncertainty and also work toward reducing the hierarchical structure.

## Conclusions

In conclusion, the study findings identified an overarching theme “ACP for Nurses in LTCHs: Power and Authority Dynamics,” which encompassed four smaller themes: (1) lacking clarity about ACP, (2) uncertainty of the nursing role in ACP, (3) feeling uncomfortable engaging in ACP discussions with residents and families, and (4) struggling to support families in ACP discussions. This study highlighted the urgent need for LTCH organizations to tackle barriers hindering the engagement of RNs and RPNs in ACP, to better support the QOL at EOL of residents. The implementation of education strategies, and development of communication guidelines for nurses can reduce the perceived hierarchical structure. It is crucial to empower nurses to enhance their engagement in ACP, ensuring the values and preferences of residents regarding future care are conveyed to all stakeholders effectively.

## Supplemental Material

sj-docx-1-son-10.1177_23779608241249335 - Supplemental material for Exploring the Role of Nurses in Advance Care Planning Within Long-Term Care Homes: A Qualitative Study Supplemental material, sj-docx-1-son-10.1177_23779608241249335 for Exploring the Role of Nurses in Advance Care Planning Within Long-Term Care Homes: A Qualitative Study by Harveer Punia, Sharon Kaasalainen, Jenny Ploeg, Patricia Strachan and Tamara Sussman in SAGE Open Nursing

sj-docx-2-son-10.1177_23779608241249335 - Supplemental material for Exploring the Role of Nurses in Advance Care Planning Within Long-Term Care Homes: A Qualitative Study Supplemental material, sj-docx-2-son-10.1177_23779608241249335 for Exploring the Role of Nurses in Advance Care Planning Within Long-Term Care Homes: A Qualitative Study by Harveer Punia, Sharon Kaasalainen, Jenny Ploeg, Patricia Strachan and Tamara Sussman in SAGE Open Nursing
