# Supplementary material for: Exploring the Role of Nurses in Advance Care Planning Within Long-Term Care Homes: A Qualitative Study
Source: SAGE Open Nurs. 2024 Apr 29;10:23779608241249335. doi: 10.1177/23779608241249335 (PMC11060033; doi:10.1177/23779608241249335)
Supplement: sj-docx-1-son-10.1177_23779608241249335 - Supplemental material for Exploring the Role of Nurses in Advance Care Planning Within Long-Term Care Homes: A Qualitative Study [file sj-docx-1-son-10.1177_23779608241249335.docx]

Semi-Structured Interview Guide

November 2018

**RN/RPN: INTERVIEW GUIDE**

Document: Interview guide targeted towards Registered Nurses and Registered Practical Nurses

Used by: Primary Researcher

Purpose: The purpose of this study is to explore experiences and perceptions of long-term care (LTC) nurses caring for individuals diagnosed with life-limiting chronic illnesses, in respect to their role in engaging in ACP.

**Introduction**

Hi _____, My name is (name), I am a Researcher at McMaster University. I am contacting you about a study that is being done by McMaster University to explore experiences and perceptions of LTC nurses in respect to their role in facilitating ACP.

I believe you have already spoken with ____ from ______ and gave permission for me to contact you and do a brief interview.

***Depending on response***

I want to start by saying thank you for agreeing to participate in this interview today. I do understand some questions during this interview may be difficult and emotional topics. Please only share what you feel comfortable sharing and tell me if you prefer not to say anymore on a topic I have asked you about.

Go over:

- Consent (review and sign)
- Demographic Form
- Address for gift card
- Begin Recording
- This study has been reviewed by the Hamilton Integrated Research Ethics Board under project #5707

**Background Questions**

1. Based on your experiences, what are your thoughts regarding advance care planning in the LTC setting?

a. What do you know about advance care planning? o What things would you want to know more about?

b. Why do you think can help facilitate advance care planning in your setting?

c. What are some of the things that make facilitating advance care planning difficult? Why?

2. How do you feel that you currently engage with your clients with chronic life limiting illnesses, when it comes to advance care planning?

a. Do you think you are working to your full capacity to engage in advance care planning?

**Role Specific Questions**

1. Can you describe your role in advance care planning is?

a. Do you think you have a role?

b. Can you describe a scenario where you engaged in ACP?

2. Do you think other staff (nurses, social workers, doctors) have a role in advance care planning?

a. Describe their role?

b. How do you collaborate with them? Can you describe a scenario where you did?

3. What kinds of activities do you think in a perfect world can help you facilitate ACP to your best potential?

a. How do you think these activities would be useful to your patients?

b. What barriers do you anticipate?

c. Do you have suggestions of how to overcome barriers?

4. Do you think it would be beneficial to have more information regarding your role and scope when it comes to advance care planning?

a. What specific things would you want more training about?

5. Is there anything else that you would like to share about your experience with advance care planning in respect to your role?
